# The “Tri-Glide” Technique: A Case Report on a Novel Intraoperative Approach for Removal of Retained and Encrusted Ureteral Stents

**DOI:** 10.1155/2022/5708348

**Published:** 2022-02-03

**Authors:** Alejandra Perez, Adam Carl Nolte, Giuseppe Maurici, Alexander Charles Small, Spencer Steve Liem, Jorge Francisco Pereira, Alan Scott Polackwich, Rafael Yanes, Ojas Shah

**Affiliations:** ^1^Mount Sinai Medical Center, Department of Urology, 4302 Alton Road, MSOP Suite 540, Miami Beach, FL 33140, USA; ^2^Olympus Medical Systems Corp., 4302 Alton Road, MSOP Suite 540, Miami Beach, FL 33140, USA; ^3^Montefiore Medical Center, 1250 Waters Place, Bronx, NY 10461, USA; ^4^Columbia University Irving Medical Center, Department of Urology, 161 Fort Washington Ave., New York, NY 10032, USA

## Abstract

**Background:**

Retained ureteral stents can result in significant morbidity and can be surgically challenging to urologists. A multimodal approach is often necessary for removal, potentially including retrograde and antegrade procedures performed over multiple anesthetic sessions. We describe the novel “Tri-Glide” technique for treating retained stents, particularly those with stent shaft encrustation prohibiting safe removal. *Case Presentation*. Two patients with nephrolithiasis and retained, encrusted ureteral stents were managed with the “Tri-Glide” technique. Patient #1 was a 58-year-old man with a severely calcified ureteral stent, retained for 14 years. After undergoing simultaneous cystolitholapaxy and percutaneous nephrolithotomy to treat proximal and distal encrustations, the stent shaft remained trapped in the ureter due to heavy calcifications. Three hydrophilic guidewires were passed alongside the stent, allowing it to easily slide out of the ureter intact. Patient #2 was a 74-year-old man who after only 3-months of stent dwell time developed severe stent shaft encrustation preventing removal. After multiple maneuvers failed, the “Tri-Glide” technique was used to create a smooth track for stent to slide out intact with gentle traction. Both patients did well postoperatively with no complications.

**Conclusion:**

The “Tri-Glide” technique can aid in the management of complex encrusted stent extractions, especially when there is significant shaft encrustation.

## 1. Introduction

Since their first description in 1967, ureteral stents remain one of urologists' most commonly used tools for relief of renal and ureteral obstruction [1]. Regardless of the indication for stent placement, patient education, clear postoperative instructions, and proper record-keeping to coordinate removal are imperative to prevent prolonged stent retention. Long-term retained stents can lead to pain, encrustation, obstruction, urinary tract infections (UTI), and ultimately loss of kidney function. Encrustation is a multifactorial process involving biofilm formation, patient risk factors (e.g., stone history, UTI, and pregnancy), characteristics of individual stent materials, and most importantly duration of stent dwell time with some studies demonstrating exponential encrustation rate over time [1].

Retained and encrusted ureteral stents can lead to significant morbidity, sometimes requiring multiple complex procedures for removal [1]. In this report, we describe a novel intraoperative technique to aid in the removal of these stents. The “Tri-Glide” technique involves passage of three hydrophilic guidewires adjacent to a stent, creating a hydrophilic track and allowing a stent to slide out with decreased friction. This approach is particularly useful for extraction of an encrusted stent body after other procedures such as cystolitholapaxy and/or percutaneous nephrolithotomy (PCNL) are used for large stone burdens at the proximal and/or distal coils of the stent. We demonstrate that in two patients, the “Tri-Glide” technique led to shortened operative times, fewer procedures, and decreased patient morbidity.

## 2. Cases

### 2.1. Patient #1: Antegrade “Tri-Glide” Technique

**Figure 1:**
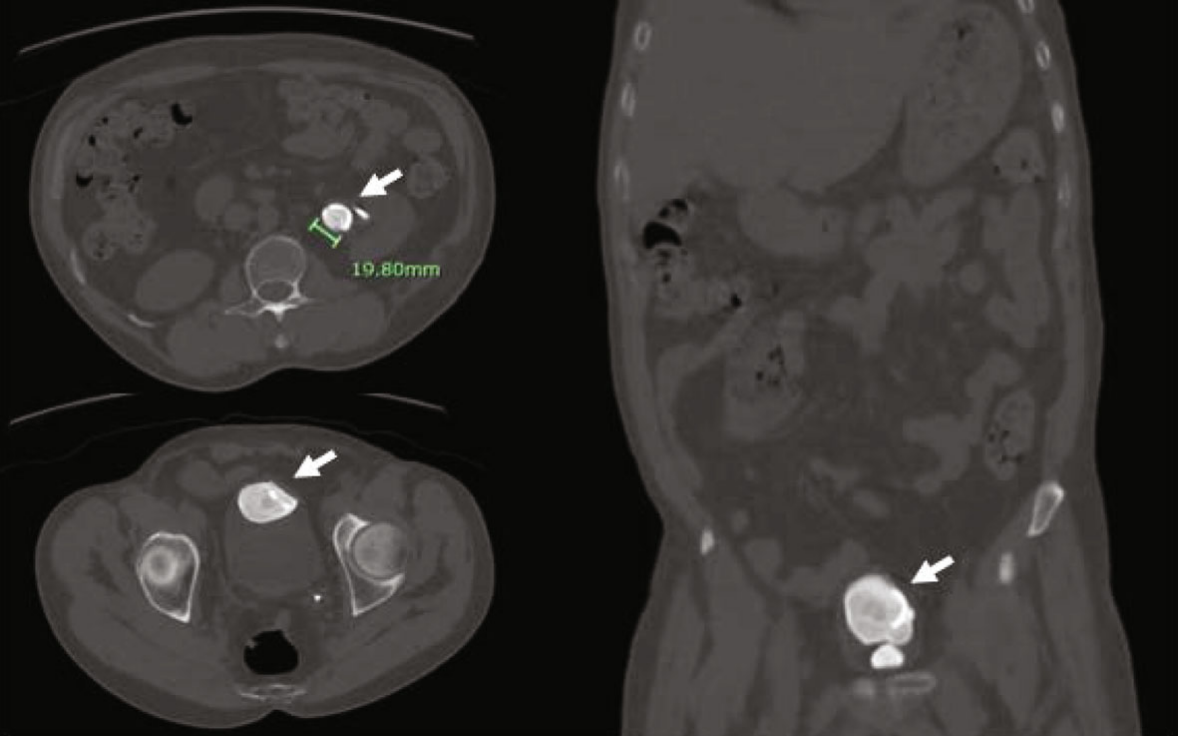
Patient 1 preoperative CT scan showing severe encrustation of the left ureteral stent at the proximal and distal coils (arrows).

The first patient was a 58-year-old man found to have a retained left ureteral stent placed fourteen years prior for an episode of nephrolithiasis. On presentation, the patient had severe left-sided hydronephrosis and large proximal and distal encrustations encasing the stent (Figure 1). He first underwent nephrostomy tube placement, followed by cystolitholapaxy and left percutaneous nephrolithotomy (PCNL) in a Galdakao-modified Valdivia supine position to allow access to the bladder and kidney simultaneously. A 0.038^″^ Boston Scientific hybrid guidewire was initially placed as a safety wire. Once the proximal and distal ends of the stent were freed from the extensive encrustation, gentle traction was applied with a grasper through the nephroscope in an effort to remove the stent shaft through the PCNL tract. However, the stent was immobile and remained fixed in place. At this point, three 0.035^″^ Terumo hydrophilic guidewires were each advanced in an antegrade fashion adjacent to the stent until they were visualized in the bladder under fluoroscopy, and the hybrid guidewire was removed. On a second attempt to withdraw the encrusted stent, it slid out of the ureter easily and was removed intact. The shaft was noted to be severely encrusted. The ureter was cleared of residual stone fragments, and the procedure was concluded.

### 2.2. Patient #2: Retrograde “Tri-Glide” Technique

**Figure 2:**
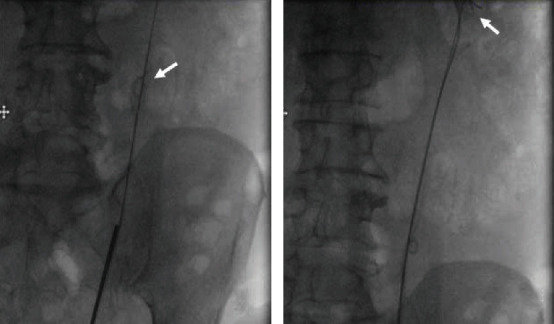
Intraoperative fluoroscopy images from patient #2. (a) The coiled stent in the midureter where it became lodged (arrow). The ureteroscope was unable to be advanced beyond the midureter. (b) 3 hydrophilic guidewires successfully passed to the kidney (arrow), at which point the stent was removed intact.

The second patient was a 74-year-old man with a long history of nephrolithiasis and recurrent UTIs requiring multiple surgical interventions, which led to development of a nonfunctional right kidney. Most recently, he had undergone left ureteroscopy with laser lithotripsy and stent placement for treatment of multiple renal calculi. Three months later, he presented for a second stage ureteroscopy and laser lithotripsy. During the procedure, a 0.038^″^ Boston Scientific hybrid guidewire was advanced to the kidney alongside the stent as a safety wire; however, the stent was unable to be completely extracted using gentle traction. The proximal coil was noted to be stuck in the midureter on fluoroscopy (Figure 2(a)). Semirigid ureteroscopy adjacent to the stent revealed the stent shaft to be severely encrusted. Ureteroscopy with laser lithotripsy was attempted to remove the encrustation, but the ureteroscope could not be safely advanced to the site of the proximal stent coil in the midureter. Therefore, the “Tri-Glide” technique was employed in a retrograde fashion—three 0.035^″^ Terumo hydrophilic guidewires were advanced alongside the stent until they were visualized in the kidney (Figure 2(b)). The hybrid guidewire was removed, and the encrusted stent was then removed easily and intact using gentle traction with a cystoscopic grasper.

## 3. Discussion

The removal of retained and encrusted ureteral stents can be surgically challenging, and various multimodal approaches can be employed to render patients stent and stone free. Combinations of percutaneous nephrolithotomy, shockwave lithotripsy, ureteroscopy with laser lithotripsy, cystolitholapaxy, and open surgical removal constitute endourologists' armamentarium for treating encrusted stents. The “Tri-Glide” technique is a successful new maneuver to add to this toolbox.

Despite the frequency of this problem, the available evidence for the optimal treatment of retained stents is primarily comprised of case reports and small series. Although no widely accepted guidelines exist, various authors have proposed algorithmic approaches for successful stent removal, which include degree of stent encrustation, stone size, and location. A classification system for forgotten, encrusted, and calcified (“FECal”) Double J stents was proposed by Acosta-Miranda et al. which describes the degree of stent encrustation (linear or bulky), stone size, and location in an algorithmic approach to guide treatment [2]. Murthy et al. also suggested an algorithm for forgotten, encrusted ureteral stents based on renal function, presence of infection, kidney salvageability, and finally, stone burden and site of encrustation as seen on imaging [3]. Generally, if there is lower coil calcification, cystolitholapaxy is indicated, while retrograde or antegrade ureteroscopy is performed for shaft encrustation [3]. If there is minimal upper coil encrustation, SWL is suggested, but if a large volume of proximal coil calcification exists, PCNL and antegrade ureteroscopy would likely be the next step [3]. Using the FECal stent classification, patient #1 would reflect a grade V level of encrustation with calcification found throughout the entirety of the stent. However, patient #2, who had primarily stent shaft encrustation with minimal distal or proximal coil calcifications, falls outside of the criteria for the FECal ureteral stent grading system and could potentially represent a rare, but important new category.

Retained stents often require multiple endourological procedures for removal with several series reporting averages of 2 to 4.2 procedures to render patients stent and stone free [3]. However, multiple single-step approaches have also been successful. These primarily describe the use of retrograde ureteroscopy with Holmium-YAG laser to treat stent calcifications along the way and in some instances fragment the stent itself in order to create more space in the ureter for the instruments. In a retrospective 2017 study, He et al. demonstrated that utilizing a smaller caliber ureteroscope 4.5/6.5 F can be effective in cases where the ureteral orifice or body cannot accommodate a larger, 8/9.8 F ureteroscope. In their series of 36 patients, ureteroscopy was found to be successful in removing encrusted stents in a single anesthetic session [4]. Use of a smaller ureteroscope can lead to reduction in the risk of mucosal injury, ureteral perforation, and ureteral avulsion as well as preclude the need for PCNL which has higher morbidity and complications [4].

Even with success of a ureteroscopic approach in multiple series, a percutaneous approach will be necessary in patients with large proximal coil stone burden and concomitant large renal stones. Indeed, the degree of proximal loop encrustation has been found to correlate with need for PCNL, multiple procedures, and risk of surgical complications [5]. Pais et al. published a multicenter retrospective study of 38 renal units that underwent PCNL for removal of encrusted ureteral stents with a mean dwell time of 28.2 months [5]. They concluded that PCNL alone was only sufficient in 21% of cases, and adjunctive procedures are often required at the time of PCNL or as a separate operation for stent removal [5].

The evolution in positioning for percutaneous nephrolithotomy has led to a shift towards prone and supine modifications that allow for simultaneous antegrade and retrograde access. In our series, a Galdakao-modified Valdivia supine position was utilized in patient 1 to allow two surgeons to work simultaneously to free the proximal and distal coils, thereby decreasing operative time. This can also be performed in a prone split-leg fashion to allow simultaneous access. In our patient, the 0.035^″^ Terumo hydrophilic guidewires were passed through the nephroscope in an antegrade fashion. However, the use of modified positioning would allow for the guidewires to be passed either retrograde or antegrade in the event of severe stent shaft encrustation, which is another advantage of the “Tri-Glide” technique.

Risks of retrograde ureteroscopy with laser lithotripsy include stent migration, ureteric injury, and perforation [3]. The use of the “Tri-Glide” technique can aid in the management of complex encrusted stent extractions, specifically when there is significant shaft encrustation or when the coils cannot be uncurled completely. It is a straightforward option in cases where a ureteroscope is unable to be advanced next to the stent secondary to severe encrustation or ureteral narrowing.

## 4. Conclusion

Encrustation and retention of ureteral stents can be a serious complication often requiring multiple procedures and a multimodal approach. During difficult extractions due to stent shaft calcification, we describe the novel “Tri-Glide” technique using three hydrophilic guidewires passed simultaneously adjacent to the stent to create a passage for an encrusted stent to be removed from the ureter in a single step.
